# Hypofractionated Versus Conventional Postmastectomy Radiotherapy in Implant-Based Breast Reconstruction: A Systematic Review and Meta-Analysis

**DOI:** 10.3390/jcm15145732

**Published:** 2026-07-22

**Authors:** Ji Hyeon Joo, Yongkan Ki, Youn-Joo Jung, Hyun Yul Kim, Ki Seok Choo, Kyung Jin Nam, Su Bong Nam

**Affiliations:** 1 Department of Radiation Oncology, Pusan National University School of Medicine, Pusan National University Yangsan Hospital, Yangsan 50612, Republic of Korea; hi_juji@daum.net (J.H.J.);; 2 Department of Surgery, Pusan National University Yangsan Hospital, Yangsan 50612, Republic of Korea; 3 Department of Surgery, Pusan National University School of Medicine, Yangsan 50612, Republic of Korea; 4 Department of Radiology, Pusan National University School of Medicine, Pusan National University Yangsan Hospital, Yangsan 50612, Republic of Korea; 5 Department of Plastic and Reconstructive Surgery, Pusan National University School of Medicine, Pusan National University Yangsan Hospital, Yangsan 50612, Republic of Korea

**Keywords:** implant-based breast reconstruction, hypofractionated postmastectomy radiotherapy, radiation-related reconstructive complications, infection, reconstructive failure

## Abstract

**Background/Objectives:** The safety of hypofractionated postmastectomy radiotherapy (HF-PMRT) in patients undergoing implant-based breast reconstruction remains uncertain because of concerns regarding radiation-related reconstructive complications. We conducted a systematic review and meta-analysis to compare reconstruction-related complications following HF-PMRT versus conventionally fractionated (CF) PMRT. **Methods:** PubMed, Cochrane Library, and EMBASE databases were searched through August 2025 following PRISMA guidelines. Studies comparing HF-PMRT with CF-PMRT in patients undergoing implant-based breast reconstruction were included. Outcomes were capsular contracture, implant or tissue expander removal, infection, and wound dehiscence. Risk of bias was assessed using RoB 2 and ROBINS-I tools. Odds ratios (ORs) with 95% confidence intervals (CIs) were pooled using the Mantel–Haenszel method. Subgroup analyses were performed by reconstruction stage (tissue expander vs. permanent implant). **Results:** Seven studies (*n* = 2200 patients; 1153 HF, 1047 CF) were analyzed, comprising two randomized controlled trials and five retrospective cohort studies. HF-PMRT was associated with a significantly lower risk of capsular contracture (OR 0.65, 95% CI 0.47–0.92) and wound dehiscence (OR 0.39, 95% CI 0.16–0.94) than CF-PMRT. Implant or tissue expander removal (OR 0.90, 95% CI 0.67–1.22) or infection (OR 0.98, 95% CI 0.67–1.44) did not differ. In subgroup analyses, reduction in capsular contracture was most evident when PMRT was delivered during the tissue expansion phase. **Conclusions:** These findings suggest that moderate hypofractionation is a reasonable fractionation option for implant-based breast reconstruction, with no evidence of increased reconstructive complications, although the certainty of evidence was limited, and prospective validation is warranted.

## 1. Introduction

Breast reconstruction has become an integral component of modern breast cancer care, with an increasing proportion of patients undergoing immediate or staged reconstruction at the time of mastectomy. In parallel, radiation therapy (RT) remains a cornerstone of locoregional management, contributing to improved disease control and survival in selected patients [1]. As indications for postmastectomy RT (PMRT) have expanded, a growing number of patients with implant-based breast reconstruction now receive PMRT, raising concerns regarding reconstruction-related complications and long-term aesthetic outcomes.

Over the past decade, hypofractionated (HF) RT has emerged as a standard approach in breast cancer treatment. Large randomized phase III trials with long-term follow-up have consistently demonstrated that moderate hypofractionation provides equivalent oncologic outcomes with comparable or improved toxicity profiles compared with conventional fractionation (CF) in the adjuvant setting [2,3,4,5,6]. Consequently, hypofractionation has been adopted in routine practice; the use of HF schedules for adjuvant breast irradiation has increased from less than 20–30% in the early 2010s to over 70–80% in recent years [7]. Building on this evidence, HF regimens are now increasingly being used for regional nodal irradiation and PMRT, supported by recent randomized trial evidence in the postmastectomy setting [3,8,9].

Implant-based reconstruction is the most commonly performed reconstructive modality worldwide due to its relative technical simplicity and broad eligibility [10]. However, RT is a well-established risk factor for adverse reconstructive outcomes, including capsular contracture, infection, wound complications, and reconstruction failure. Prior evidence indicates higher complication rates following PMRT in implant-based reconstruction compared with autologous reconstruction [11]. Given the larger dose per fraction in hypofractionated regimens, there is a theoretical concern that hypofractionation may exacerbate radiation-induced fibrosis and increase the risk of capsular contracture or implant loss, making the application of HF-PMRT in this population an area of clinical uncertainty. Furthermore, the reconstruction stage at the time of irradiation (tissue expander versus permanent implant) appears to modify complication risk, underscoring the importance of stage-specific analyses when evaluating outcomes [12].

Although several retrospective cohort studies have investigated reconstruction-related complications following HF PMRT, their findings have been inconsistent and frequently limited by small sample sizes and heterogeneous patient populations [13,14,15]. Importantly, recently reported randomized phase III trials now provide an opportunity to more rigorously reassess reconstruction-related safety in the postmastectomy setting [16,17]. Therefore, we conducted a systematic review and meta-analysis comparing reconstruction-related complications following HF versus CF PMRT in patients undergoing implant-based breast reconstruction. We focused specifically on moderate hypofractionation (2.4–2.7 Gy per fraction, e.g., 40.05 Gy in 15 fractions or 42.56 Gy in 16 fractions); ultra-hypofractionated five-fraction schedules, such as the 26 Gy/5-fraction FAST-Forward regimen, represent a distinct fractionation category and were outside the scope of this review. Although a larger meta-analysis of immediate reconstruction has recently been reported [18], our review focuses on photon-based, implant-based reconstruction and, by incorporating contemporary randomized evidence with a dedicated subgroup analysis by reconstruction stage, aims to clarify the safety profile of HF-PMRT and inform multidisciplinary decision-making.

## 2. Materials and Methods

### 2.1. Search Strategy and Data Sources

This review and meta-analysis were reported in accordance with the Preferred Reporting Items for Systematic Reviews and Meta-Analyses (PRISMA) 2020 guidelines (File S1) [19]. This review was not registered in a prospective registry, and no formal protocol was prepared. A comprehensive literature search was performed across three electronic databases—PubMed, Cochrane Library, and EMBASE—from 2014 through August 2025. The search strategy was designed to identify studies comparing HF with CF in patients who underwent implant-based breast reconstruction and PMRT. The search terms combined concepts of mastectomy (mastectom*, skin-sparing mastectomy, nipple-sparing mastectomy); breast reconstruction (reconstruct*, implant, expander*, prosthetic, breast reconstruction, mammaplasty); radiation fractionation (hypofractionation, hypofractionated, fractionation, IMRT, intensity-modulated radiation therapy, VMAT, volumetric-modulated arc therapy, PMRT, postmastectomy radiotherapy); and complications (complication*, capsular contracture, explant*, implant loss, infection, wound dehiscence, failure, toxicity). Both Medical Subject Headings (MeSH) terms and free-text keywords were utilized. The complete search strings for each database are provided in Table S1.

### 2.2. Study Selection and Data Extraction

Studies were included based on the following criteria: (1) patients underwent prosthetic (implant-based) reconstruction after mastectomy for breast cancer; (2) the study population included patients receiving both HF PMRT and CF PMRT; (3) reconstruction-related complication outcomes were reported; and (4) full-text articles were published in English. Exclusion criteria were: (a) reviews, case reports, comments, editorials, letters, surveys, and books; (b) ongoing clinical trials without published results; (c) duplicate publications; and (d) studies not reporting separate outcomes for HF and CF groups. Two authors independently screened the titles and abstracts. Potentially eligible studies underwent full-text assessment. Extracted data included: first author, publication year, country, study design, patient demographics, sample size for each treatment arm, radiation dose and fractionation schedules, reconstruction type at PMRT (tissue expander vs. permanent implant), follow-up duration, and complication outcomes.

The primary outcomes of interest were reconstruction-related complications, including capsular contracture (Baker grades ≥3 or equivalent), implant loss/explantation, infection requiring intervention, and wound dehiscence. For studies that included both tissue expander and permanent implant reconstructions, outcome data were assigned to a reconstruction-stage subgroup only when the original study reported event counts separately by reconstruction stage; where stage-specific counts were not directly reported, the study contributed to the overall analysis only, and no subgroup data were derived or imputed.

### 2.3. Risk of Bias and Certainty of Evidence Assessment

The Risk of Bias 2 (RoB 2) tool was applied to randomized controlled trials (RCTs) [20], whereas non-randomized studies were appraised with Risk of Bias in Non-Randomized Studies of Interventions (ROBINS-I) [21]. The certainty of evidence for key outcomes was determined using the Grading of Recommendations Assessment, Development and Evaluation (GRADE) framework [22,23]. Given the inclusion of both RCTs and retrospective cohort studies, evidence from RCTs was prioritized, while data from retrospective cohort studies were incorporated when RCT certainty was moderate or low, following GRADE guidance for integrating randomized and non-randomized evidence.

### 2.4. Statistical Analysis

Complication rates were compared between HF and CF groups as binary outcomes, expressed as odds ratios (ORs) with 95% confidence intervals (CIs). The Mantel–Haenszel method was selected as the primary pooling approach due to its robustness in handling sparse data and small sample sizes, which are common characteristics in reconstruction complication studies. Statistical heterogeneity was evaluated using Cochran’s Q test and the I^2^ statistic. A fixed-effect model was pre-specified as the primary approach. Statistical heterogeneity was quantified using Cochran’s Q test and the I^2^ statistic, and a random-effects (DerSimonian–Laird) model was applied as a sensitivity analysis for all outcomes.

Studies reporting zero events in both treatment arms were excluded from the quantitative synthesis, as effect estimates cannot be reliably calculated using the Mantel–Haenszel method. When zero cells occurred in one arm only, a continuity correction of 0.5 was added to all cells of the 2 × 2 table to enable OR calculation for that study. Subgroup analyses were performed stratified by reconstruction stage (tissue expander vs. permanent implant). The significance of differences between subgroups was assessed using a Q-test for subgroup interaction. Two-tailed *p*-values < 0.05 were considered statistically significant. All meta-analyses were conducted using R statistical software (version 4.5.2; R Foundation for Statistical Computing, Vienna, Austria) with the metafor package (version 4.8.0). Publication bias was not formally assessed using funnel plots or Egger’s test, as fewer than 10 studies were available for each outcome, the recommended minimum for reliable assessment [24].

## 3. Results

### 3.1. Study Selection and Characteristics

**Figure 1:**
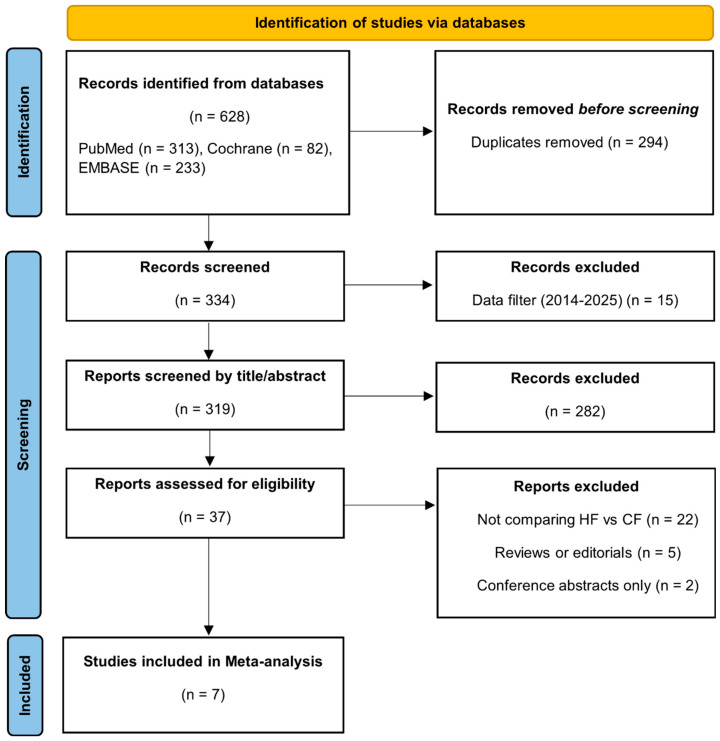
Preferred Reporting Items for Systematic Reviews and Meta-Analyses (PRISMA) flow diagram of study selection. HF: hypofractionation; CF: conventional fractionation.

The initial database search yielded 628 records (PubMed: 313, Cochrane: 82, EMBASE: 233). After removing 294 duplicates, 334 records remained. Following application of the date filter (2014–2025), the remaining 319 records underwent title and abstract screening, with 282 articles excluded based on the screening criteria. The remaining 37 articles underwent full-text assessment for eligibility, and 30 were excluded for various reasons. Ultimately, seven studies met all inclusion criteria and were included in the meta-analysis (Figure 1). Included studies published between 2019 and 2025 are summarized in Table 1. Four studies, two studies, and one study were conducted in Korea, the United States, and China, respectively. Study designs comprised two RCTs (FABREC trial and subgroup analysis of FDRT-BC008) [16,17] and five retrospective cohort studies, including nationwide, multicenter, and single-center designs [15,25,26,27,28]. A total of 2200 patients with implant-based breast reconstruction followed by PMRT were included. Of these, 1153 (52.4%) and 1047 (47.6%) were in the HF and CF groups, respectively. The HF protocols delivered 40.05–46.3 Gy over 14–24 fractions (2.4–2.7 Gy per fraction), while CF protocols administered 45–50.4 Gy over 25–35 fractions (1.8–2.0 Gy per fraction). Follow-up duration ranged from 17.9 to 40.4 months across studies.

### 3.2. Risk of Bias and GRADE Assessment

**Figure 2:**
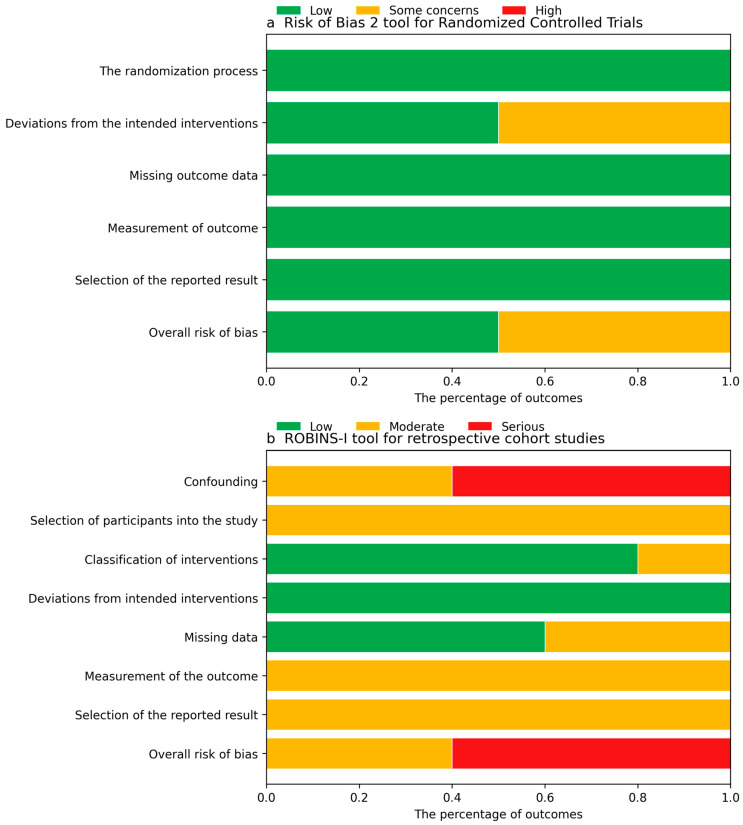
Risk of bias assessment for the two included randomized controlled trials and the five retrospective cohort studies.

Risk of bias in the two included RCTs and the five retrospective studies was assessed, with 50% and 60% of outcomes, respectively, rated as having some concerns or serious risk of bias (Figure 2). Full assessments for RCTs (RoB 2) and retrospective studies (ROBINS-I) are provided in Tables S2 and S3. According to the GRADE assessment, for outcomes informed by RCT evidence, the certainty of evidence was rated as low for implant or tissue expander removal, infection, and wound dehiscence, and very low for capsular contracture. The primary reason for the lower rating was imprecision related to limited event numbers, with additional downgrading for risk of bias in some outcomes. For outcomes informed by retrospective studies, the certainty of evidence was consistently rated as very low across all outcomes. Full details of the GRADE assessment are presented in Table S4.

### 3.3. Meta-Analysis of Reconstruction-Related Complications

**Figure 3:**
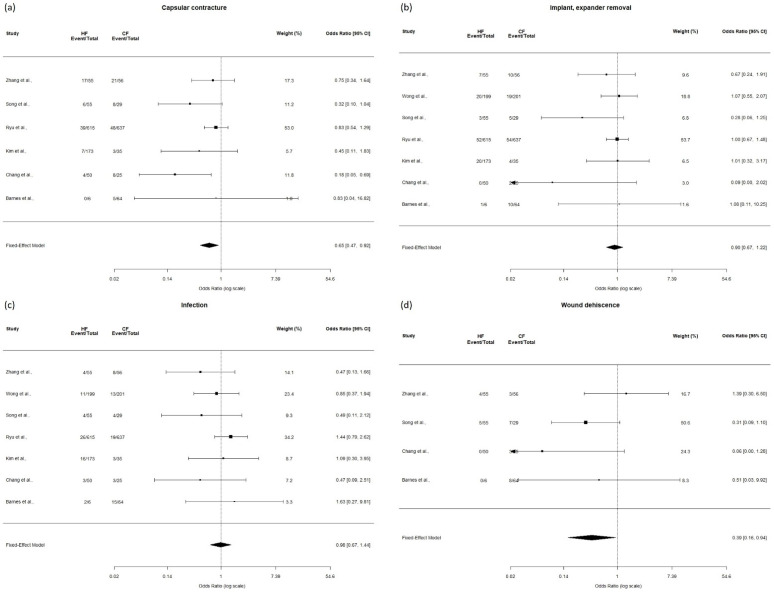
Forest plots of odds ratios comparing hypofractionation versus conventional fractionation: (**a**) capsular contracture, (**b**) implant or tissue expander removal, (**c**) infection, and (**d**) wound dehiscence. Included studies: Zhang et al. [17], Wong et al. [16], Song et al. [28], Ryu et al. [15], Kim et al. [27], Chang et al. [26], and Barnes et al. [25]. HF: hypofractionation; CF: conventional fractionation; CI: confidence interval.

HF was associated with significantly reduced capsular contracture compared to CF (OR: 0.65; 95% CI: 0.47–0.92; *p* = 0.015; *n* = 6) (Figure 3a). Six studies (900 HF patients, 887 CF patients) reported capsular contracture across all reconstruction types [15,17,25,26,27,28]. Between-study heterogeneity was low (I^2^ = 12%, *p* = 0.34). For implant or tissue expander removal, no significant difference was observed between HF and CF groups (OR: 0.90; 95% CI: 0.67–1.22; *p* = 0.51; *n* = 7) (Figure 3b). All seven studies (1153 HF patients, 1047 CF patients) provided data on the outcome [15,16,17,25,26,27,28]. Heterogeneity remained minimal across studies (I^2^ = 0%, *p* = 0.92). For infection, no statistically significant difference was detected between treatment groups (OR: 0.98; 95% CI: 0.67–1.44; *p* = 0.92; *n* = 7) (Figure 3c). All seven studies (1153 HF patients, 1047 CF patients) examined infection rates requiring intervention [15,16,17,25,26,27,28]. Between-study heterogeneity was absent (I^2^ = 0%, *p* = 0.78). HF was associated with significantly reduced wound dehiscence (OR: 0.39; 95% CI: 0.16–0.94; *p* = 0.036; *n* = 4) (Figure 3d). Four studies (166 HF patients, 174 CF patients) reported wound dehiscence complications, and no heterogeneity was detected among the studies (I^2^ = 0%, *p* = 0.87) [17,25,26,28].

In a random-effects sensitivity analysis, results were consistent with the primary analysis for most outcomes (Table S5). The reduction in capsular contracture remained significant (OR 0.59, 95% CI 0.37–0.92), while infection (OR 0.98, 95% CI 0.67–1.45) and implant or tissue expander removal (OR 0.91, 95% CI 0.67–1.23) remained non-significant. For wound dehiscence, however, the association was no longer significant (OR 0.45, 95% CI 0.15–1.35), indicating that this finding was not robust to the choice of model. Results stratified by study design were broadly concordant, although pooling of the randomized evidence was not possible for capsular contracture or wound dehiscence, as only a single trial reported each (Table S6).

### 3.4. Subgroup Analysis by Reconstruction Stage

**Figure 4:**
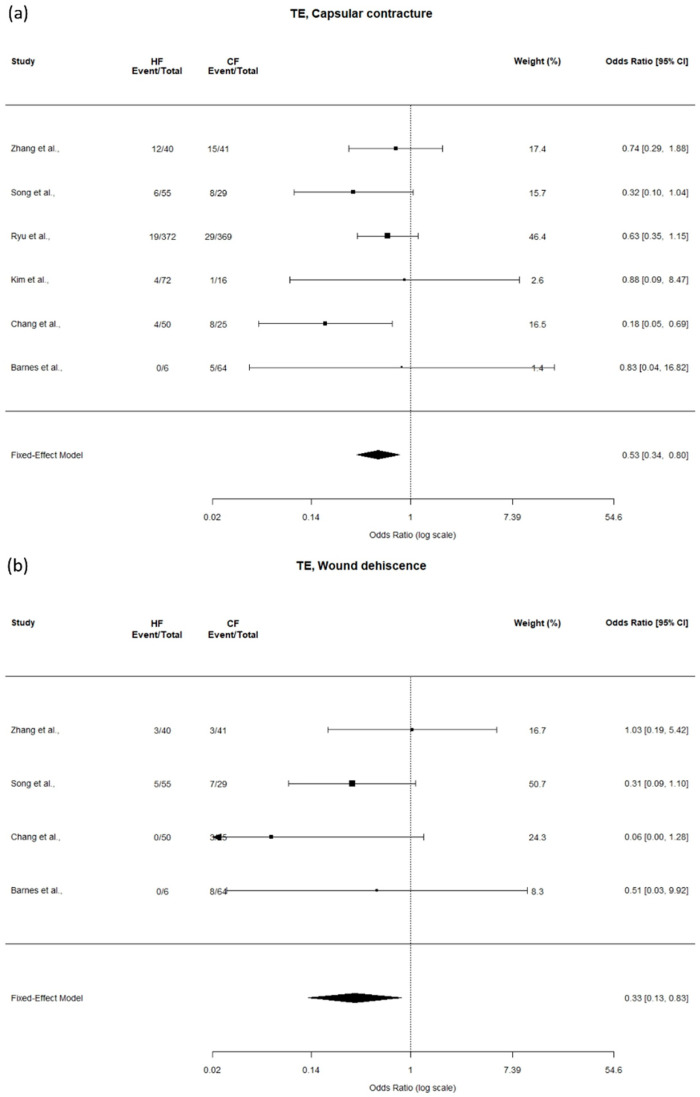
Forest plots of odds ratios comparing hypofractionation versus conventional fractionation in patients undergoing tissue expander–based reconstruction: (**a**) capsular contracture and (**b**) wound dehiscence. Included studies: Zhang et al. [17], Song et al. [28], Ryu et al. [15], Kim et al. [27], Chang et al. [26], and Barnes et al. [25]. HF: hypofractionation; CF: conventional fractionation; CI: confidence interval.

Six studies (595 patients: 295 HF, 300 CF) reported outcomes for tissue expander reconstruction, representing patients who underwent PMRT during the tissue expansion phase [15,17,25,26,27,28]. HF was associated with a significantly lower odds of capsular contracture compared to CF (OR: 0.53; 95% CI: 0.34–0.80; *p* = 0.003; *n* = 6) (Figure 4a) [15,17,25,26,27,28]. Between-study heterogeneity was low (I^2^ = 0%, *p* = 0.68). HF did not increase the rate of expander or implant removal (OR: 0.84; 95% CI: 0.57–1.25; *p* = 0.39; *n* = 6, I^2^ = 0%, *p* = 0.88) or rate of infection (OR: 1.12; 95% CI: 0.67–1.88; *p* = 0.66; *n* = 6, I^2^ = 0%, *p* = 0.94) [15,17,25,26,27,28]. HF demonstrated significantly reduced wound dehiscence (OR: 0.33; 95% CI: 0.13–0.83; *p* = 0.019; *n* = 4) (Figure 4b), with no heterogeneity detected (I^2^ = 0%, *p* = 0.75) [17,25,26,28].

Three studies (661 patients: 359 HF, 302 CF) specifically examined permanent implant reconstruction, representing patients who underwent RT on the implant [15,17,27]. The pooled estimate showed no significant difference in capsular contracture incidence between HF and CF groups (OR: 0.98; 95% CI: 0.55–1.73; *p* = 0.94; *n* = 3) [15,17,27]. Between-study heterogeneity was absent (I^2^ = 0%, *p* = 0.53). No significant difference was observed in infection rates (OR: 0.77; 95% CI: 0.33–1.77; *p* = 0.54; *n* = 3), and heterogeneity was minimal (I^2^ = 0%, *p* = 0.45) [15,17,27]. The rate of implant removal revealed no significant difference between HF and CF groups (OR: 0.88; 95% CI: 0.45–1.71; *p* = 0.70; *n* = 3), with no heterogeneity detected (I^2^ = 0%, *p* = 0.71) [15,17,27]. A formal subgroup interaction test did not demonstrate a statistically significant difference in capsular contracture reduction between the two reconstruction stages (Q = 2.80, df = 1, *p* = 0.094).

## 4. Discussion

As survival outcomes for patients with breast cancer continue to improve, greater emphasis is being placed on ensuring that PMRT does not worsen the outcomes of implant-based breast reconstruction. Minimizing radiation-related reconstructive morbidity has become an essential clinical objective in HF-PMRT adoption, underscoring the importance of a refined understanding of radiation-induced tissue effects and close multidisciplinary collaboration.

Although autologous reconstruction is associated with superior aesthetic outcomes and satisfaction, it is often not feasible owing to patient comorbidities, insufficient donor tissue, or higher donor-site morbidity [29,30,31,32,33,34]. Implant-based reconstruction therefore remains the most commonly performed technique [35,36], making its interaction with PMRT clinically important.

Complications of implant-based reconstruction are diverse and often overlapping rather than occurring as isolated entities. Multiple systematic reviews and meta-analyses have consistently demonstrated an increased risk of major reconstruction-related complications associated with PMRT [37,38,39,40,41,42]. Among these, capsular contracture is the most significant long-term complication, with PMRT increasing its risk approximately 4.5–5-fold [37,38]. Its pathogenesis is multifactorial: radiation promotes fibroblast-to-myofibroblast transformation and fibrosis, compounded by chronic inflammation and biofilm-related immune responses [43,44,45,46,47,48]. Although contemporary series suggest an overall incidence often below 10%, this rises to approximately 19% after PMRT [49], underscoring the clinical relevance of strategies—such as fractionation choice—that may mitigate this risk.

Irradiated tissues show compromised vascular integrity and impaired immune response, increasing infection risk [50]. After PMRT, infection rates in implant-based reconstruction reach 16–21% within two years, compared with 4–13% in non-irradiated patients [11,12], and infection frequently necessitates revisional surgery or explantation [37,39,40,41].

Radiation-induced microvascular damage and fibrosis significantly impair wound healing, increasing the incidence of skin necrosis and wound breakdown—particularly when RT is delivered to a tissue expander rather than a permanent implant. In a large cohort, patients receiving RT to a tissue expander experienced higher rates of skin necrosis (10.3%), wound breakdown (9.5%), and infection (16.4%) compared to those irradiated after direct-to-implant reconstruction (skin necrosis 4.0%, wound breakdown 2.7%, infection 4.0%) [12]. Delayed wound healing has been identified as a strong predictor of reconstructive failure, with reported ORs for implant loss reaching as high as 17.86 [51]. The risk of implant removal due to wound complications is greatest within the first 3 years following RT [52].

Late-responding tissues such as fibroblasts and connective tissue are known to be sensitive to fraction size, raising concerns that hypofractionation could increase radiation-induced fibrosis and adversely affect cosmetic outcomes. However, large, randomized trials have consistently shown that moderate hypofractionation results in similar or even lower rates of late toxicity, with equivalent or improved cosmetic outcomes compared with CF [2,4,5,53]. In a pooled analysis of 11 studies (*n* = 3611), Hayashi et al. reported that moderate HF following immediate reconstruction did not raise the overall rate of major reconstructive complications relative to CF (OR 0.83, 95% CI 0.51–1.35); infection and reconstructive failure were likewise comparable between schedules, whereas capsular contracture showed a possible reduction (OR 0.38, 95% CI 0.15–0.99) [18]. These findings are reinforced by randomized evidence from the FABREC trial, which demonstrated non-inferior patient-reported outcomes and comparable late toxicity profiles following HF PMRT in implant-based reconstruction [16]. Early results from the Alliance A221505 (RT-CHARM) trial, which randomized nearly 900 patients, suggest that HF-PMRT after breast reconstruction achieves similar rates of major complications, providing prospective confirmation of the growing evidence supporting hypofractionation in this population [54]. A plausible mechanistic explanation for these findings likely reflects a combination of radiobiological and technical factors. Moderate hypofractionation delivers a lower biologically effective dose to late-responding tissues compared with CF when calculated with an α/β ratio of 3 Gy, which may attenuate radiation-induced fibrosis. In addition, contemporary HF regimens are typically planned with improved dose homogeneity within reconstructed tissues, potentially limiting high-dose regions that predispose to wound complications and implant-related adverse events. Earlier dosimetric work indicated that near-maximum dose to the reconstructed breast is governed chiefly by the total prescribed dose rather than the dose per fraction [26]. A subsequent multicenter study of 314 patients supported this dose–response pattern: contracture risk rose with increasing equivalent dose (OR per Gy, 1.58; *p* < 0.001) irrespective of reconstruction type, and HF-treated patients had fewer major contractures [55].

The timing of PMRT relative to tissue expander or permanent implant placement may influence complication patterns, although results have been heterogeneous [56]. Pooling 11 studies (*n* = 1447), Guo et al. found that irradiating a tissue expander, rather than a permanent implant, carried a higher risk of implant loss (RR 1.75, 95% CI 1.03–2.98) yet a markedly lower risk of capsular contracture (RR 0.47, 95% CI 0.29–0.78) [57]. Consistently, Lee and Mun (*n* = 900) observed that irradiation at the expander stage lowered the risk of severe capsular contracture versus permanent-implant irradiation (RR 0.44; *p* < 0.001), although overall complications tended to be more frequent [56]. In general, PMRT delivered to a tissue expander followed by implant exchange has shown an association with higher overall reconstructive complications, including infection, wound breakdown, skin necrosis, and reconstructive failure. In contrast, higher capsular contracture rates after irradiation of a permanent implant may reflect the limited opportunity to remove or remodel radiation-induced fibrotic tissue once the permanent implant is in place. Additionally, patients irradiated at the tissue expander stage may undergo capsulotomy during implant exchange, which could partially mitigate established fibrosis. However, not all studies demonstrate a clear advantage to either approach. A Mastectomy Reconstruction Outcomes Consortium study across 11 institutions found no significant differences in overall complication rates or reconstructive failure between tissue expander and permanent implant irradiation strategies [58]. Similarly, Yoon et al. reported no significant differences in satisfaction, physical well-being, or aesthetic outcomes between timing strategies in a multicenter prospective analysis [59].

In this meta-analysis, HF-PMRT was associated with a lower risk of capsular contracture and wound dehiscence compared with CF, without increased infection or reconstructive failure rates. Notably, the apparent benefit of hypofractionation was most pronounced when PMRT was delivered to a tissue expander rather than to a permanent implant; however, this difference did not reach statistical significance on formal interaction testing (*p* = 0.094) and should be considered hypothesis-generating.

Several limitations warrant consideration. The certainty of evidence for most outcomes was low to very low according to the GRADE criteria, largely due to imprecision from limited event numbers and predominance of retrospective studies. This review was also not prospectively registered, and no formal protocol was established in advance, which should be considered when interpreting our findings. Follow-up durations were relatively short, potentially underestimating the long-term incidence of capsular contracture, which may manifest several years post-treatment. Furthermore, heterogeneity in radiation techniques, implant characteristics, and acellular dermal matrix (ADM) use may have influenced the observed outcomes and limited identification of optimal treatment parameters. In contemporary practice, ADM provides additional soft tissue support and may mitigate inflammatory and fibrotic responses, potentially reducing the risk of capsular contracture and wound-related complications. However, the extent of ADM utilization varies significantly across institutions and geographic regions, and most included studies did not adequately account for its use. This may limit the generalizability of our findings and partially explain discrepancies between studies. Finally, capsular contracture was ascertained inconsistently across studies: most used the Baker classification, whereas one nationwide study defined it by capsulectomy. In addition, adjunct procedures such as capsulotomy or lipofilling were not reported in a way that allowed their influence on this outcome to be assessed. These differences may modestly underestimate its incidence and contribute to between-study heterogeneity.

## 5. Conclusions

In conclusion, this meta-analysis provides a focused synthesis of reconstructive outcomes following HF-PMRT in patients undergoing implant-based breast reconstruction, with particular attention to the reconstruction stage at the time of irradiation. Hypofractionation appears to have a favorable reconstructive safety profile, particularly regarding capsular contracture and wound complications, without increasing infection or reconstructive failure risk, although the certainty of evidence remains limited. These findings support the use of moderate hypofractionation in this setting, consistent with current guideline recommendations. However, they also highlight the need for prospective studies with longer follow-up to refine patient selection and treatment strategies.

## Figures and Tables

**Table 1:** Study characteristics for the included randomized controlled trials and retrospective cohort studies.

First Author (Year), Country	Study Design	Follow-Up, Months	No. of Patients (PI/TE)		Radiation Dose, Gy (fx)		Primary Aim	Key Results
			CF	HF	CF	HF		
Zhang (2025), China [17]	RCT (FDRT-BC008)	38.1/39.5	56 (15/41)	55 (15/40)	50 (25)	42.56 (16)	Reconstruction complications and oncologic outcomes	No increased risk with HF across TE and PI; TE had the highest complication rate
Wong (2024), USA [16]	RCT (FABREC)	40.4	201 (45/156)	199 (39/160)	50 (25)	42.56 (16)	Quality of life at 6 months after PMRT	HF improved QoL in patients ≤45 years; no association with chest-wall toxicity
Song (2020), Korea [28]	RCS	34.8	29 (TE only)	55 (TE only)	50.4 (28)	40.05 (15)	Safety of HF-VMAT	HF-VMAT reduced radiation-related morbidity
Ryu (2024), Korea [15]	RCS, population-based	~30	637 (268/369)	615 (243/372)	45–50 (25–35)	40–42.5 (14–24)	Reconstruction complications by fractionation	No difference in surgery-requiring complications between HF and CF
Kim (2022), Korea [27]	RCS, multicenter	35.3	35 (19/16) ^†^	173 (101/72) ^†^	50.4 (28)	46.3 (15–18)	Any major breast-related complications	HF did not increase complications and reduced capsular contracture
Chang (2019), Korea [26]	RCS, single-center	32.5	25 (TE only)	50 (TE only)	50.4 (28)	40.05 (15)	Dose–response relationship and complications	HF reduced near-Dmax, associated with fewer complications
Barnes (2024), USA [25]	RCS, single-center	17.9	64 (TE only)	6 (TE only)	50 (25)	42.56 (16)	Impact of modifiable RT factors	No evidence HF increases risk; dose homogeneity more critical than fractionation

CF, conventional fractionation; fx, fractions; HF, hypofractionation; PI, permanent implant; PMRT, postmastectomy radiotherapy; QoL, quality of life; RCS, retrospective cohort study; RCT, randomized controlled trial; TE, tissue expander; VMAT, volumetric-modulated arc therapy. Follow-up is shown as median months. Patient numbers are given as total (PI/TE). ^†^ Patient numbers for Kim (2022) [27] reflect the implant-based reconstruction subgroup.

## Data Availability

The original contributions presented in this study are included in the article/Supplementary Materials. Further inquiries can be directed to the corresponding author.

## Supplementary Materials

The following supporting information can be downloaded at: https://www.mdpi.com/article/10.3390/jcm15145732/s1, Table S1: Complete search strings used for each electronic database; Table S2: Reasons for the judgment of risk of bias by the Risk of Bias 2 (RoB 2) tool for randomized controlled trials; Table S3: Reasons for the judgment of risk of bias by the Risk of Bias in Non-randomized Studies of Interventions (ROBINS-I) tool for retrospective cohort studies; Table S4: Certainty of evidence according to the GRADE approach; Table S5: Sensitivity analysis using a random-effects model, compared with the primary fixed-effect analysis; Table S6: Sensitivity analysis stratified by study design (randomized controlled trials vs. retrospective cohort studies). File S1: PRISMA 2020 Checklist.
