# Supplementary material for: Hypofractionated Versus Conventional Postmastectomy Radiotherapy in Implant-Based Breast Reconstruction: A Systematic Review and Meta-Analysis
Source: J Clin Med. 2026 Jul 22;15(14):5732. doi: 10.3390/jcm15145732 (PMC13413347; doi:10.3390/jcm15145732)
Supplement: Supplementary file 1 [file jcm-15-05732-s001.zip › jcm-4379384-supplementary tables.pdf]

Supplementary Information (SI)

Title: Hypofractionated Versus Conventional Postmastectomy Radiotherapy in Implant-Based Breast Reconstruction: A Systematic Review And Meta-Analysis

Ji Hyeon Joo [1], Yongkan Ki [1], Youn Joo Jung, MD [2], Hyun Yul Kim [2,3], Ki Seok Choo [4], Kyung Jin Nam [4], and Su Bong Nam [5]

[1]Department of Radiation Oncology, Pusan National University School of Medicine, Pusan National University Yangsan Hospital, Yangsan, Republic of Korea

[2]Department of Surgery, Pusan National University Yangsan Hospital, Yangsan, Republic of Korea

[3]Department of Surgery, Pusan National University School of Medicine, Yangsan, Republic of Korea

[4]Department of Radiology, Pusan National University School of Medicine, Pusan National University Yangsan Hospital, Yangsan, Republic of Korea

[5]Department of Plastic and Reconstructive Surgery, Pusan National University School of Medicine, Pusan National University Yangsan Hospital, Yangsan, Republic of Korea

Corresponding author: Su Bong Nam, MD, PhD

Department of Plastic and Reconstructive Surgery, Pusan National University Yangsan Hospital, 20, Geumo-ro, Mulgeum-eup, Yangsan, Gyeongsangnam-do, Republic of Korea

Tel: 82-55-360-2572

Fax: 82-55-360-2158

E-mail: subong71@hanmail.net

Table S1. Complete search strings used for each electronic database.

Database	Search string
**PubMed**	((mastectom*[tiab] OR "skin-sparing mastectomy"[tiab] OR "nipple-sparing mastectomy"[tiab] OR "mastectomy"[mesh]) AND (reconstruct*[tiab] OR implant[tiab] OR expander*[tiab] OR prosthetic[tiab] OR "breast reconstruction"[tiab] OR "reconstructed breast"[tiab] OR "mammaplasty"[mesh]) AND (hypofractionation[tiab] OR hypofractionated[tiab] OR fractionation[tiab] OR imrt[tiab] OR "intensity-modulated radiation therapy"[tiab] OR vmat[tiab] OR "volumetric-modulated arc therapy"[tiab] OR pmrt[tiab] OR "postmastectomy radiotherapy"[tiab] OR "post-mastectomy radiotherapy"[tiab] OR "dose fractionation, radiation"[mesh] OR "radiation dose hypofractionation"[mesh] OR "radiotherapy, intensity-modulated"[mesh]) AND (complication*[tiab] OR "capsular contracture"[tiab] OR explant*[tiab] OR "implant loss"[tiab] OR infection[tiab] OR "wound dehiscence"[tiab] OR failure[tiab] OR toxicity[tiab]))
**Cochrane Library**	#1 (mastectom* OR "skin-sparing mastectomy" OR "nipple-sparing mastectomy"):ti,ab,kw<br>#2 (reconstruct* OR implant OR expander* OR prosthetic OR "breast reconstruction" OR "reconstructed breast"):ti,ab,kw<br>#3 (hypofractionation OR hypofractionated OR fractionation OR imrt OR "intensity-modulated radiation therapy" OR vmat OR "volumetric modulated arc therapy" OR pmrt OR "postmastectomy radiotherapy" OR "post-mastectomy radiotherapy"):ti,ab,kw<br>#4 MeSH descriptor: [Dose Fractionation, Radiation] explode all trees<br>#5 MeSH descriptor: [Radiation Dose Hypofractionation] explode all trees<br>#6 MeSH descriptor: [Radiotherapy, Intensity-Modulated] explode all trees<br>#7 #3 OR #4 OR #5 OR #6<br>#8 #1 AND #2 AND #7
**EMBASE**	(mastectom*:ti,ab OR 'skin sparing mastectomy':ti,ab OR 'nipple sparing mastectomy':ti,ab OR 'mastectomy'/exp) AND (reconstruct*:ti,ab OR implant*:ti,ab OR expander*:ti,ab OR prosthetic*:ti,ab OR 'breast reconstruction':ti,ab OR 'reconstructed breast':ti,ab OR 'mammaplasty'/exp) AND (hypofractionation:ti,ab OR hypofractionated:ti,ab OR fractionation:ti,ab OR imrt:ti,ab OR 'intensity modulated radiotherapy':ti,ab OR vmat:ti,ab OR 'volumetric modulated arc therapy':ti,ab OR pmrt:ti,ab OR 'postmastectomy radiotherapy':ti,ab OR 'post-mastectomy radiotherapy':ti,ab OR 'dose fractionation'/exp OR 'radiation dose hypofractionation'/exp OR

	'intensity modulated radiotherapy'/exp) AND (complication*:ti,ab OR 'capsular contracture':ti,ab OR explant*:ti,ab OR 'implant loss':ti,ab OR infection:ti,ab OR 'wound dehiscence':ti,ab OR failure:ti,ab OR toxicity:ti,ab)

Table S2. Reasons for the judgment of risk of bias by the Risk of Bias 2 (RoB 2) tool for randomized controlled trials.

Outcomes (study, outcome)	Risk of bias arising from the randomization process	Risk of bias due to deviations from the intended interventions	Risk of bias due to missing outcome data	Risk of bias in measurement of outcome	Risk of bias in selection of the reported result	Overall risk of bias
Wong et al. (2024, FABREC) [1] – Major chest wall toxic effects (grade ≥3: infection, delayed wound healing, TE/implant removal, unplanned surgical intervention)	Low risk	Low risk	Low risk	Low risk	Low risk	Low risk
	Computer-generated random sequence, central web-based randomization; baseline characteristics well balanced.	Open-label trial but delivered fractionation per assignment with very few crossovers; analyses essentially according to randomized groups; no evidence that deviations or co-interventions differed by arm.	Chest wall toxic effects were collected for essentially all treated patients in the as-treated cohort; no indication of substantial loss to follow-up for toxicity outcomes.	Outcomes are objective and data for all analyzed participants were reported.	Trial protocol registered; toxic effects pre-specified as a key safety endpoint; no evidence that results were selected from multiple unreported analyses.	

Zhang et al. (2025, FDRT-BC008 subgroup) [2] – Any reconstruction complication (infection, wound dehiscence, fat/skin necrosis, seroma/hematoma, capsular contracture, implant/TE removal, conversion to autologous)	Low risk	Some concerns	Low risk	Low risk	Low risk	Some concerns
	Phase III randomized design; 1:1 allocation to fractionation; baseline characteristics in the subgroup well balanced.	Open-label; three randomized patients withdrew consent after randomization and were excluded from analysis. Exclusions are few and balanced.	Among the 133 analyzed patients, follow-up for complications appears complete; the paper does not describe important loss to follow-up or differential missingness of complication data.	Outcomes are objective and data for all analyzed participants were reported.	Trial registered (NCT03856372) with predefined endpoints; complications reported comprehensively by reconstruction type and fractionation; no evidence of selective outcome reporting.	

Table S3. Reasons for the judgment of risk of bias by the Risk of Bias in Non-randomized Studies of Interventions (ROBINS-I) tool for retrospective cohort studies.

**Study**	**Bias due to confounding**	**Bias in selection of participants into the study**	**Bias in classification of interventions**	**Bias due to deviations from intended interventions**	**Bias due to missing data**	**Bias in measurement of outcomes**	**Bias in selection of the reported result**	**Overall risk of bias**
	**Serious risk**	**Moderate risk**	**Low risk**	**Low risk**	**Low risk**	**Moderate risk**	**Moderate risk**	**Serious risk**
**Song *et al.* (2020) [3]**	Treatment indication/fractionation likely associated with baseline disease and treatment factors; residual confounding expected.	Single-institution retrospective cohort with potential selection bias despite defined inclusion criteria.	Treatment groups were clearly defined according to radiotherapy technique and fractionation.	No evidence of outcome-related deviations from intended interventions.	Complication data were available for the entire cohort with no major loss to follow-up reported.	Outcomes were abstracted from medical records; outcome assessors were not blinded.	Lack of prespecified protocol raises the possibility of selective reporting.	
	**Moderate risk**	**Moderate risk**	**Low risk**	**Low risk**	**Low risk**	**Moderate risk**	**Moderate risk**	**Moderate risk**
**Chang *et al.* (2019) [4]**	Multivariable adjustment included key patient factors (eg, age, BMI, smoking) and showed adjusted association between	Retrospective single-center design with potential selection bias.	Radiotherapy dose and dosimetric parameters were clearly defined.	No evidence of deviations related to outcomes.	Outcome data were complete for all included patients.	Complications were assessed by two plastic surgeons; lack of blinding may still allow	Absence of a prespecified analysis plan raises concerns regarding	

	dose metrics and complications; residual/unmeasured confounding remains possible.					assessment bias.	selective reporting.	
	**Moderate risk**	**Moderate risk**	**Low risk**	**Low risk**	**Low risk**	**Moderate risk**	**Moderate risk**	**Moderate risk**
**Kim *et al.* (2022) [5]**	Multivariable adjustment addressed several key confounders; however, residual confounding due to treatment era and technique differences remained possible.	Two-institution retrospective cohort with potential selection bias in exposure groups.	Hypofractionated and conventional regimens were clearly defined.	No evidence of outcome-related deviations after treatment initiation.	Exclusion criteria and follow-up were clearly described with minimal missing data.	Outcomes were derived from medical records; definitions were clinically clear but subject to reporting variability.	Retrospective design without protocol registration allows potential selective reporting.	
	**Serious risk**	**Moderate risk**	**Moderate risk**	**Low risk**	**Moderate risk**	**Moderate risk**	**Moderate risk**	**Serious risk**
**Ryu *et al.* (2024) [6]**	Propensity score adjustment reduced confounding; however, key clinical variables were unavailable in claims data.	Selection was based on administrative claims, which may not fully capture clinical eligibility.	Fractionation was inferred from claims codes, allowing potential exposure misclassification.	Delivered radiotherapy was captured through claims without evidence of outcome-related deviation.	Claims data minimize loss to follow-up, but under-ascertainment of events is possible.	Complications were identified using diagnosis and procedure codes, which may vary in sensitivity and specificity.	Prespecified analyses were reported, but administrative data limit outcome granularity.	

	**Serious risk**	**Moderate risk**	**Low risk**	**Low risk**	**Moderate risk**	**Moderate risk**	**Moderate risk**	**Serious risk**
**Barnes *et al.* (2024) [7]**	Analyses largely based on bivariable models, providing limited control for confounding.	Single-center retrospective design with potential selection bias.	Radiotherapy fractionation and technique were objectively recorded.	Analyses reflected delivered treatment without evidence of outcome-driven deviations.	Short follow-up duration may have limited detection of late complications.	Chart-based outcome assessment without blinding may introduce assessment bias.	Reporting focused on statistically significant associations among exploratory analyses.	

Table S4. Certainty of evidence according to the GRADE approach.

**Outcome**	**Included studies**	**Starting level**	**Risk of bias**	**Inconsistency**	**Indirectness**	**Imprecision**	**Publication bias**	**Overall certainty (GRADE)**
**Capsular contracture**	RCTs (1)	High	Serious[1]	Serious[2]	Not serious	Serious[3]	Suspected[4]	**Very low**
	RCSs (5)	Low	Serious[1]	Serious[2]	Not serious	Serious[3]	Suspected[4]	**Very low**
**Implant / tissue expander removal**	RCTs (2)	High	Serious[1]	Not serious	Not serious	Serious[3]	Not suspected	**Low**
	RCSs (5)	Low	Serious[1]	Not serious	Not serious	Serious[3]	Not suspected	**Very low**
**Infection**	RCTs (2)	High	Serious[1]	Not serious	Not serious	Serious[3]	Not suspected	**Low**
	RCSs (5)	Low	Serious[1]	Not serious	Not serious	Serious[3]	Not suspected	**Very low**
**Wound dehiscence**	RCTs (1)	High	Serious[1]	Not serious	Not serious	Serious[3]	Not suspected	**Low**
	RCSs (3)	Low	Serious[1]	Not serious	Not serious	Serious[3]	Not suspected	**Very low**

[1] Downgraded one level for risk of bias due to limitations in study design, including retrospective cohorts and some concerns in randomized evidence.

[2] Downgraded one level for inconsistency due to variability in effect estimates across studies.

[3] Downgraded one level for imprecision due to small number of events and wide confidence intervals.

[4] Downgraded one level for suspected publication bias.

Table S5. Sensitivity analysis using a random-effects model, compared with the primary fixed-effect analysis.

Outcome	No. of studies	Primary analysis (fixed-effect)[1]			Sensitivity analysis (random-effects)[2]			
		**OR**	**95% CI**	***p***	**OR**	**95% CI**	***p***	**I^2 (%)[3]**
Capsular contracture	6	0.65	0.47–0.92	0.013	0.59	0.37–0.92	0.021	23
Infection	7	0.98	0.67–1.44	0.923	0.98	0.67–1.45	0.929	0
Wound dehiscence	4	0.39	0.16–0.94	0.035	0.45	0.15–1.35	0.156	25
Implant/TE removal	7	0.90	0.67–1.22	0.505	0.91	0.67–1.23	0.549	0

OR, odds ratio; CI, confidence interval; I^2, Higgins inconsistency statistic. Odds ratios <1 favour hypofractionation (fewer events).

[1] Pooled using the fixed-effect model (Mantel–Haenszel), pre-specified as the primary analysis.

[2] Pooled using a random-effects model (DerSimonian–Laird), applied as a pre-planned sensitivity analysis for all outcomes.

[3] Between-study heterogeneity quantified using Cochran's Q test and the I^2 statistic.

Table S6. Sensitivity analysis stratified by study design (randomized controlled trials vs retrospective cohort studies).

**Outcome**	**Study design**	**No. of studies**	**OR (95% CI)[1]**	***p***	**I^2 (%)**
Capsular contracture[2]	RCT	1	0.75 (0.34–1.64)	—	—
	RCS	5	0.49 (0.26–0.93)	0.030	37
Infection	RCT	2	0.71 (0.35–1.42)	0.331	0
	RCS	5	1.14 (0.71–1.83)	0.580	0
Wound dehiscence[2]	RCT	1	1.39 (0.30–6.50)	—	—
	RCS	3	0.27 (0.09–0.80)	0.018	0
Implant/TE removal	RCT	2	0.94 (0.54–1.64)	0.819	0
	RCS	5	0.82 (0.48–1.39)	0.450	16

OR, odds ratio; CI, confidence interval; I^2, Higgins inconsistency statistic; RCT, randomized controlled trial; RCS, retrospective cohort study. Odds ratios <1 favour hypofractionation (fewer events).

[1] Pooled within each stratum using a random-effects (DerSimonian–Laird) model.

[2] Only a single randomized trial (Zhang et al.) reported this outcome; the value shown is the individual study estimate, as pooling was not possible (the FABREC trial did not report capsular contracture).

References

1 Wong, J. S.; Uno, H.; Tramontano, A. C.; Fisher, L.; Pellegrini, C. V.; Abel, G. A.; Burstein, H. J.; Chun, Y. S.; King, T. A.; Schrag, D.; et al. Hypofractionated vs Conventionally Fractionated Postmastectomy Radiation After Implant-Based Reconstruction: A Randomized Clinical Trial. *JAMA Oncol* **2024**, *10*, 1370–1378. https://doi.org/10.1001/jamaoncol.2024.2652

2 Zhang, X.; Wang, X.; Chu, Y.; Zhang, L.; Meng, J.; Shi, W.; Chen, X.; Yang, Z.; Mei, X.; Yu, X.; et al. Post-mastectomy hypofractionated versus conventionally fractionated radiation therapy for patients receiving immediate breast reconstruction: Subgroup analysis of a phase III randomized trial. *Clin Transl Radiat Oncol* **2024**, *50*, 100882. https://doi.org/10.1016/j.ctro.2024.100882

3 Song, S. Y.; Chang, J. S.; Fan, K. L.; Kim, M. J.; Chang, H. P.; Lew, D. H.; Roh, T. S.; Roh, H.; Kim, Y. B.; Lee, D. W. Hypofractionated Radiotherapy With Volumetric Modulated Arc Therapy Decreases Postoperative Complications in Prosthetic Breast Reconstructions: A Clinicopathologic Study. *Front Oncol* **2020**, *10*, 577136. https://doi.org/10.3389/fonc.2020.577136

4 Chang, J. S.; Song, S. Y.; Oh, J. H.; Lew, D. H.; Roh, T. S.; Kim, S. Y.; Keum, K. C.; Lee, D. W.; Kim, Y. B. Influence of Radiation Dose to Reconstructed Breast Following Mastectomy on Complication in Breast Cancer Patients Undergoing Two-Stage Prosthetic Breast Reconstruction. *Front Oncol* **2019**, *9*, 243. https://doi.org/10.3389/fonc.2019.00243

5 Kim, D. Y.; Park, E.; Heo, C. Y.; Jin, U. S.; Kim, E. K.; Han, W.; Shin, K. H.; Kim, I. A. Influence of Hypofractionated Versus Conventional Fractionated Postmastectomy Radiation Therapy in Breast Cancer Patients With Reconstruction. *Int J Radiat Oncol Biol Phys* **2022**, *112*, 445–456. https://doi.org/10.1016/j.ijrobp.2021.09.031

6 Ryu, H.; Shin, K. H.; Chang, J. H.; Jang, B. S. A nationwide study of breast reconstruction after mastectomy in patients with breast cancer receiving postmastectomy radiotherapy: comparison of complications according to radiotherapy fractionation and reconstruction procedures. *Br J Cancer* **2024**, *131*, 290–298. https://doi.org/10.1038/s41416-024-02741-4

7 Barnes, L. L.; Chew, J.; Lem, M.; Park, C.; Yang, J. C.; Prionas, N.; Piper, M. Modifiable Postmastectomy Radiation Therapy Factors and Impact on Implant-Based Breast Reconstruction Outcomes. *Plast Reconstr Surg* **2024**, *153*, 1000–1009. https://doi.org/10.1097/PRS.0000000000010824
